# Spring-assisted cranioplasty for sagittal synostosis: long-term clinical and patient-reported outcomes: Ten-year outcomes of spring-assisted cranioplasty for isolated sagittal synostosis: a single-institution cohort study

**DOI:** 10.1007/s00381-026-07337-2

**Published:** 2026-06-12

**Authors:** Amparo Saenz, David Dunaway, Greg James, Juling Ong, Dulanka Silva, Alessandro Borghi, Silvia Schievano, Owase Jeelani

**Affiliations:** 1 Pediatric Neurosurgery Department, Great Ormond Street Hospital, London, UK; 2 Craniofacial Unit, Great Ormond Street Hospital, London, UK; 3 Department of Engineering, Durham University, Durham, DH13LE UK; 4 UCL Great Ormond Street Institute of Child Health, London, UK

**Keywords:** Craniosynostosis, Sprig cranioplasty, Scaphocepaly, Sagittal synostosis, Minimally invasive surgery

## Abstract

**Purpose:**

Spring-assisted cranioplasty is an established technique for the correction of isolated sagittal craniosynostosis, yet data on long-term clinical durability and patient-perceived outcomes remain limited. The purpose of this study was to evaluate long-term surgical, aesthetic, and patient-reported outcomes following spring-assisted cranioplasty, with a particular focus on the need for secondary cranial surgery and concordance between surgeon- and parent-reported assessments.

**Methods:**

A single-centre cohort study was performed, including consecutive patients with isolated, nonsyndromic sagittal synostosis treated with spring-assisted cranioplasty between April 2010 and September 2015. Patients were followed within a standardized multidisciplinary craniofacial pathway extending into late childhood and adolescence. Long-term outcomes were assessed using reoperation rates, Kaplan–Meier reoperation-free survival analysis, surgeon-reported Whitaker classification, and structured parent-reported satisfaction measures.

**Results:**

Ninety-one of the original 100 patients had complete long-term follow-up and were included, with a mean follow-up duration of 10.0 years (range 5–15 years). Nine patients (9.9%) required secondary cranial surgery during follow-up, including four for raised intracranial pressure and five for aesthetic indications. Reoperation-free survival was 90.1% at 10 years, with the median survival not reached. Surgeon-reported outcomes were excellent, with 87.9% classified as Whitaker Class I. Parent-reported satisfaction was high, with 82.4% completely satisfied and unwilling to consider further surgery.

**Conclusion:**

Spring-assisted cranioplasty for isolated sagittal synostosis provides durable long-term functional and aesthetic outcomes, with a low incidence of secondary cranial surgery and high patient and parent satisfaction when combined with structured multidisciplinary follow-up.

## Introduction

Spring-assisted cranioplasty was first popularised by Lauritzen and colleagues [1] in 1998 as a method of dynamic cranial reshaping, using implanted springs for the correction of sagittal craniosynostosis. The principle of active internal expansion relies on using force vectors on the inherent malleability of the paediatric skull and represents a departure from traditional static remodeling techniques. It offered a biomechanically more effective alternative to isolated suturectomy, with the potential to create controlled vault widening during a period of rapid brain growth. Since that initial description, spring-assisted approaches have gained international interest, accompanied by ongoing refinement in both technique and device design.

At Great Ormond Street Hospital for Children (GOSH), spring-assisted cranioplasty for the correction of isolated sagittal synostosis was initiated in April 2010, utilizing a novel spring design developed in-house following extensive bioengineering analysis [2–11]. This technique was adopted to achieve active cranial expansion through a standardized, minimally invasive approach while minimizing operative morbidity in young infants. Early and mid-term outcomes from this cohort have previously been reported [12], demonstrating favourable perioperative safety and effective mid-term morphologic improvement.

Spring-assisted techniques have since gained widespread adoption across multiple centres globally, and published reviews confirm that this approach can be performed safely and efficiently in appropriately selected patients [13–24]. Nevertheless, important questions persist regarding the broader clinical implications of spring-assisted correction [25]. These include the learning curve associated with the techniques, the long-term outcomes, and the balance between reduced surgical invasiveness and the need for secondary procedures, whether planned (spring removal) or unplanned (secondary cranial remodeling). In this context, comparisons with traditional remodeling procedures have often focused on early operative metrics, while longer-term clinical outcomes have remained less well defined [19, 26].

With the original GOSH cohort now reaching late childhood, a unique opportunity exists to evaluate outcomes approaching a decade after the initial spring-assisted procedure. The present study aims to provide a detailed description of our current surgical technique for sagittal spring insertion, highlighting technical nuances and refinements developed through experience, and to report long-term outcomes of the initial 100 cases that received spring-assisted cranioplasty for sagittal synostosis at our institution.

## Methods

### Study design and patient selection

This is a single-centre cohort analysis of patients with isolated, nonsyndromic sagittal craniosynostosis treated with spring-assisted cranioplasty at GOSH, London. The cohort includes consecutive patients who underwent primary spring-assisted correction of sagittal synostosis during the early implementation phase of this technique at our institution.

Patients were eligible for inclusion if they had a diagnosis of isolated sagittal craniosynostosis confirmed on clinical assessment, with or without adjunctive imaging, and were treated primarily with spring-assisted cranioplasty. Patients with syndromic craniosynostosis, multisuture involvement, or those who underwent alternative primary surgical strategies were excluded from the study. The initial surgical procedures were performed between April 2010 and September 2015, corresponding to the cohort previously reported for perioperative and mid-term outcomes [12].

All patients included in the present analysis were followed longitudinally as part of routine craniofacial surveillance within the multidisciplinary craniofacial service. Long-term follow-up data were obtained from outpatient clinic visits, electronic medical records, and the institutional craniofacial database. For the purposes of long-term outcome analysis, only patients with documented follow-up extending into late childhood were included. Patients who had been lost to long-term follow-up were excluded from the present analysis.

The original cohort has been previously described with respect to operative details, perioperative outcomes, and early morphologic changes. The current analysis builds upon that cohort by incorporating extended follow-up, evaluation of the need for secondary cranial surgery, and structured long-term outcome assessment from both surgeon and parent perspectives. Ethical approval and institutional governance approvals were obtained in accordance with local research and audit regulations.

### Operative technique

**Fig. 1:**
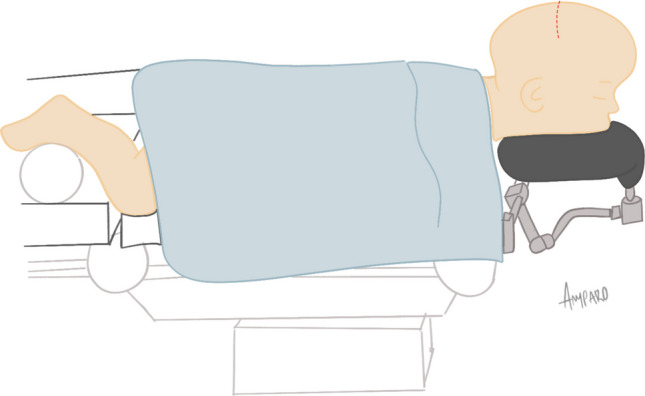
Illustration demonstrating patient positioning for spring-assisted cranioplasty. The patient is placed prone in a sphinx position with the neck gently extended and the vertex oriented parallel to the operating table. Care is taken to ensure stable head support, neutral cervical alignment, and unobstructed venous drainage throughout the procedure

Spring-assisted cranioplasty is performed under general anesthesia with the patient positioned prone in a sphinx position, with the neck extended and the top of the head oriented parallel to the operating table (Fig. 1). Care is taken to ensure stable head positioning and unobstructed venous drainage throughout the procedure.

**Fig. 2:**
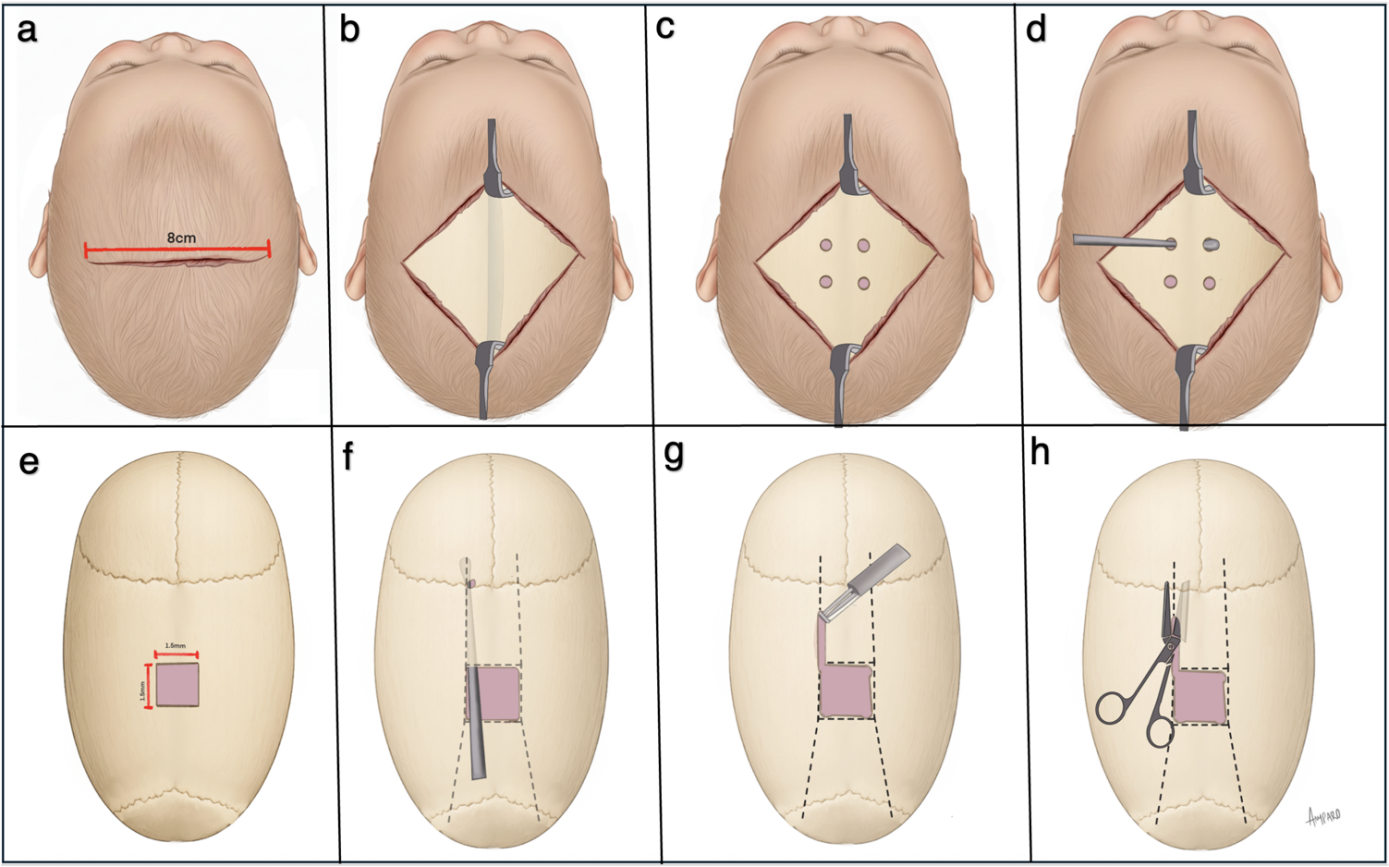
Illustrations showing the exposure and osteotomy technique for spring-assisted cranioplasty. **a** A single transverse scalp incision of approximately 8 cm is made perpendicular to the sagittal suture, positioned slightly anterior to the midpoint between the anterior and posterior fontanelles. **b** Subgaleal dissection is carried anteriorly and posteriorly to expose the sagittal suture and extend toward the coronal and lambdoid sutures as required. **c** A central 1.5 × 1.5 cm midline square is marked, and burr holes are placed at each corner. **d** Careful dissection is carried out between the burr holes to minimize the risk of dural tears during use of the craniotome. **e** Using the craniotome, a central 1.5 × 1.5 cm square midline craniectomy is performed over the sagittal sinus. **f** Careful dural dissection is performed along the planned parasagittal osteotomy lines to ensure complete separation of the dura from the inner table before bone cutting. **g** Parasagittal longitudinal osteotomies are primarily fashioned using a craniotome. **h** Bone scissors are used to complete the anterior and posterior limits of the osteotomies near the coronal and lambdoid sutures to improve tactile control and safety

A single transverse scalp incision is made perpendicular to the sagittal suture (Fig. 2a). Based on long-term scar behaviour observed during follow-up, the incision is positioned slightly anterior to the midpoint between the anterior and posterior fontanelles, as scars tend to migrate posteriorly toward the posterior vertex during skull remodelling and growth. Subgaleal dissection is carried anteriorly and posteriorly to expose the sagittal suture region, extending toward the coronal and lambdoid sutures as required (Fig. 2b).

A small square 1.5 × 1.5 cm midline craniectomy is preformed out over the sagittal sinus at approximately the midpoint of the fused sagittal suture (Fig. 2c–-e). This step has proven useful in improving safety by reducing the risk of a dural breach.

Two parasagittal longitudinal osteotomies are then fashioned, beginning from the margins of the central craniectomy and extending anteriorly toward the coronal sutures and posteriorly toward the lambdoid sutures. Before initiating these osteotomies, careful dural dissection is performed along the planned osteotomy lines to ensure complete separation of the dura from the inner table to reduce the risk for inadvertent dural breach (Fig. 2f).

**Fig. 3:**
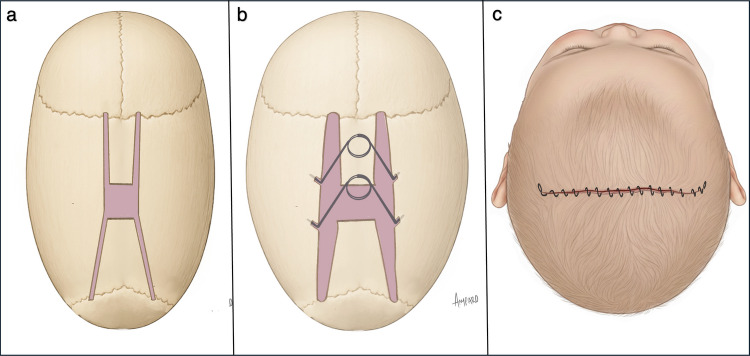
Illustrations of completion of osteotomies, spring placement, and wound closure. **a** Final configuration of the parasagittal osteotomies extending into the coronal and lambdoid sutures, with preservation of the central midline bone strut to facilitate ossification during the expansion phase. **b** Insertion of two cranial springs into the prepared lateral grooves on either side of the midline, with both spring helices oriented anteriorly and resting on intact bone to provide controlled, symmetrical expansion. Changes in the skull shape can be seen after the insertion of springs. **c** Final wound closure following placement of a subgaleal drain, using resorbable sutures along the transverse scalp incision

The osteotomies are performed primarily using a craniotome (Fig. 2g); however, as the cuts approach their anterior and posterior limits, bone scissors are utilised to complete the final cuts (Fig. 2h). This transition allows greater tactile control and improved safety when working near the coronal and lambdoid sutures. The osteotomies are extended into the coronal and lambdoid sutures. It is important to ensure this is the case, and incomplete osteotomies are likely to lead to asymmetric expansion and suboptimal results (Fig. 3a).

Throughout the procedure, particular attention is paid to dural protection, including during preparation of the lateral grooves for spring placement. The dura is gently dissected away from the inner table before fashioning the grooves with the craniotome. The central midline bone strut is preserved to facilitate subsequent ossification during the expansion phase.

Two cranial springs are inserted into the prepared grooves on either side of the midline (Fig. 3b). Based on postoperative comfort and clinical observation, both springs are positioned with the helix oriented anteriorly. Earlier configurations using opposing helix orientations were associated with discomfort in the supine position in the immediate perioperative period in some children. The spring helices are positioned to rest securely on intact bone, and care is taken to avoid placing the helix directly over the central craniectomy defect whenever possible.

Spring selection is individualized; however, with increasing experience, there has been a deliberate shift toward more gentle expansion strategies. In most cases, springs with lower stiffness provide effective and sustained cranial remodeling. The optimal spring force is considered to be the minimal elastic force required to generate progressive expansion over time rather than maximal immediate opening. On-table assessment confirms appropriate engagement and controlled separation.

A subgaleal drain is placed, and the wound is closed in layers using resorbable sutures (Fig. 3c). Postoperatively, patients are monitored in a recovery room before transfer to a standard neurosurgical ward. The drain is typically removed at 24 h, and a postoperative radiograph is obtained to confirm spring position. Discharge is usually possible on the first postoperative day.

Spring removal is performed as a planned secondary procedure, typically approximately three months after insertion. Removal is undertaken through the original incision under general anesthesia and is generally performed as a day-case procedure.

### Long-term follow-up and outcome assessment

All patients are enrolled in a standardised multidisciplinary craniofacial follow-up pathway extending to 15 years of age, with scheduled reviews at nationally agreed time points. Early and mid-childhood surveillance includes neurosurgical assessment for raised intracranial pressure, ophthalmological review, and serial three-dimensional photographic assessment. From school age, outcome assessment incorporates patient- and parent-reported measures, including formal psychological evaluation at approximately 7 years. Reviews at 10 and 15 years, led by consultant craniofacial plastic surgeons, focus on aesthetic outcome, cranial shape stability, and shared decision-making regarding secondary procedures. For the present study, outcome data were extracted from each patient’s most recent documented follow-up visit.

### Outcome measures

Long-term outcomes were assessed using a combination of objective clinical endpoints and structured surgeon- and parent-reported measures, recorded as part of routine multidisciplinary follow-up.

### Secondary cranial surgery

Secondary cranial surgery was defined as any cranial procedure performed after the initial spring-assisted cranioplasty and the planned subsequent spring removal. Secondary cranial surgery events included both early reoperations previously reported in the original cohort analysis and additional reoperations identified during extended long-term follow-up. Secondary procedures were categorized according to their primary indication as either treatment for raised intracranial pressure or correction of residual or recurrent cranial deformity for aesthetic reasons. For each patient requiring secondary surgery, the type of procedure and the interval between the index operation and secondary intervention were recorded.

### Surgeon-reported outcome

Surgeon-reported long-term outcome was assessed using the Whitaker classification [27], a widely used outcome scale in craniofacial surgery based on the recommendation for additional operative intervention. Outcomes were classified as follows: Class I, no additional surgery recommended or required; Class II, minor contouring or soft-tissue revision suggested; Class III, major cranial remodeling or osteotomies recommended; and Class IV, major craniofacial procedure equivalent in magnitude to the original operation. Whitaker classification was used to assess aesthetic and reconstructive outcome and was not applied to classify secondary surgery performed primarily for raised intracranial pressure. The Whitaker classification was assigned at the most recent follow-up visit by a consultant craniofacial surgeon.

### Parent-reported outcome

Parent-reported outcome was derived from prospectively collected clinical information recorded as part of routine multidisciplinary follow-up. At school age and beyond, parents and patients undergo structured assessment within the craniofacial follow-up pathway, including formal psychological evaluation and consultant-led craniofacial review. These assessments routinely include parental and patient perspectives on head shape, appearance-related concerns, and the perceived impact of cranial shape on daily life and psychosocial well-being.

For the purposes of the present study, parent-reported satisfaction was summarized using a four-point categorical scale reflecting overall satisfaction with head-shape outcome and willingness to consider further cranial surgery. Outcomes were classified as follows: I, completely satisfied and would not consider further head-shape surgery; II, mostly satisfied but might consider minor contouring if it were simple or low risk; III, not fully satisfied and would consider a further corrective cranial procedure if recommended; and IV, dissatisfied and actively seeking or desiring further surgery. This assessment was intended to capture parental perception of long-term aesthetic outcome and was not used to evaluate functional indications such as raised intracranial pressure.

### Data acquisition

This retrospective cohort study was conducted in accordance with the principles of the Declaration of Helsinki. Specific consent for this analysis was not required; written consent for anonymised use of clinical data for research was obtained from all families at the time of surgery. Clinical data were obtained from the institutional craniofacial database and electronic medical records, including demographics, operative dates, follow-up duration, secondary surgery events, and outcome classification. Follow-up duration was calculated from the index procedure to the most recent documented craniofacial clinic visit. Operative and perioperative data from the index procedure have been previously reported and were not reanalysed here.

### Statistical analysis

Continuous variables are presented as means with standard deviations or ranges. Categorical variables are summarized using frequencies and percentages.

Time to secondary cranial surgery was calculated from the date of the index spring-assisted cranioplasty to the date of secondary intervention, where applicable. Patients without secondary surgery were censored at the date of last follow-up. Kaplan–Meier survival analysis was performed to estimate reoperation-free survival over time, with 95% confidence intervals calculated using the Greenwood formula. Survival probabilities were estimated at key time points (1, 2, 3, 5, and 10 years postoperatively).

The relationship between surgeon-reported outcomes (Whitaker classification) and parent/patient-reported satisfaction was examined using cross-tabulation and visualized using a heatmap and stacked bar chart representations. The association between these outcome measures was assessed using a chi-square test with Monte Carlo simulation (10,000 iterations) to account for small cell counts.

All analyses were performed using R (Version 4.4.2) with RStudio (Version 2024.12.0 + 467). Statistical packages included survival (version 3.8–3) for Kaplan–Meier analysis, ggplot2 (version 3.5.1) for data visualization, and dplyr (version 1.1.4) for data manipulation. Statistical significance was set at *p* < 0.05 where applicable.

## Results

### Cohort characteristics and follow-up

Of the original 100 patients treated with spring-assisted cranioplasty, 91 had complete long-term follow-up data and were included in the present analysis. Nine patients had relocated abroad and were therefore excluded. The 91 patients consisted exclusively of those with isolated nonsyndromic sagittal synostosis, with a median age at initial surgery of 5.0 months (range 3.0–11.0), a median hospital stay of 1.4 days (range 1.0–55.5), a median age at spring removal of 8.0 months (range 4.0–19.0), and a mean follow-up duration of 10.0 years (SD 2.2, range 5.0–15.0).

### Reoperation outcomes

**Fig. 4:**
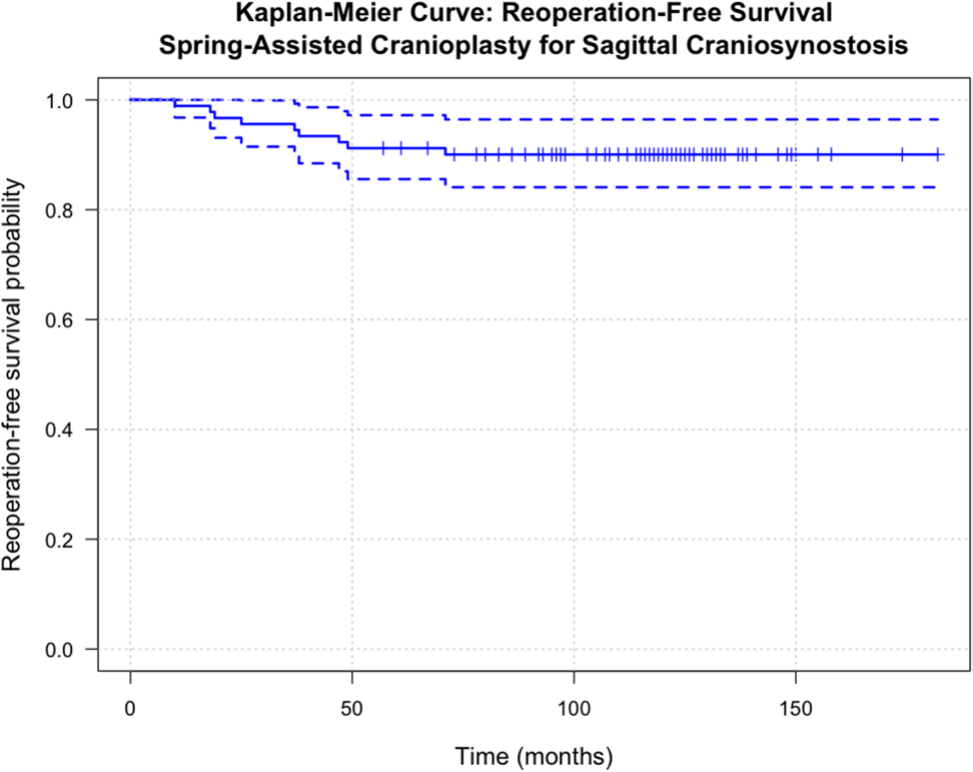
Kaplan–Meier curve showing reoperation-free survival following spring-assisted cranioplasty for sagittal craniosynostosis. The blue line represents the survival function, with the shaded area indicating 95% confidence intervals. Vertical tick marks denote censored observations (patients without reoperation at last follow-up). The median reoperation-free survival was not reached, indicating that more than 50% of patients remained reoperation-free throughout the entire follow-up period extending to 182 months (15 years). The reoperation-free survival rate was 91.2% at 5 years and 90.1% at 10 years post-initial surgery

At initial follow-up, four patients (4.4%) required early reoperation. Kaplan–Meier survival analysis was performed to assess long-term reoperation-free survival (Fig. 4). The median follow-up time for the survival analysis was 118 months (mean 109.9 months, range 10–182 months). During this period, nine patients (9.9%) required reoperation, while 82 patients (90.1%) remained reoperation-free. The median reoperation-free survival was not reached, indicating that more than 50% of patients remained free from reoperation throughout the entire follow-up period (Table 1).

**Table 1:** Kaplan–Meier survival analysis: reoperation-free survival at key time points

Time point	Time (years)	Patients at risk	Reoperation-free survival (%)	95% CI	Cumulative reoperation rate (%)	95% CI
12 months	1	90	98.9	96.8–100.0	1.1	0.0–3.2
24 months	2	88	96.7	93.1–100.0	3.3	0.0–6.9
36 months	3	87	95.6	91.5–99.9	4.4	0.1–8.5
60 months	5	82	91.2	85.6–97.2	8.8	2.8–14.4
120 months	10	43	90.1	84.1–96.4	9.9	3.6–15.9

Of the nine patients who required secondary surgery, four underwent reoperation for raised intracranial pressure and five for aesthetic reasons.

Among the four patients with raised intracranial pressure, two were previously reported in the initial cohort analysis and underwent surgery at 38 and 49 months after the index procedure. Both patients presented with papilledema and received posterior vault expansion. The two additional cases of raised intracranial pressure identified during extended follow-up required surgery at 25 and 71 months postoperatively. One patient presented with clinical symptoms of raised intracranial pressure and underwent invasive intracranial pressure monitoring, which confirmed the diagnosis; the other patient presented with papilledema. One patient underwent posterior vault expansion, while the other required total calvarial remodelling.

Five patients required secondary surgery for aesthetic indications. Two patients were previously reported in the initial analysis and underwent fronto-orbital remodelling at 18 and 19 months after the index procedure, both for frontal bossing. Three additional patients required aesthetic revision surgery at 10, 37, and 47 months postoperatively. One patient developed coronal suture fusion during follow-up, resulting in facial asymmetry that required fronto-orbital remodelling; genetic testing was negative for this patient. One patient had persistent narrow biparietal width and underwent anterior two-thirds cranial remodelling. The third patient developed frontal bossing and required fronto-orbital remodelling.

### Surgeon-reported outcomes

Long-term aesthetic and reconstructive outcomes were assessed using the Whitaker classification at the most recent follow-up visit. The majority of patients demonstrated excellent outcomes on surgeon-reported assessment, with 80 patients (87.9%) classified as Whitaker Class I (no additional surgery recommended or required), 6 patients (6.6%) as Class II (minor contouring or soft-tissue revision suggested), and 5 patients (5.5%) as Class IV (major craniofacial procedure equivalent in magnitude to the original operation). No patients were classified as Whitaker Class III.

The primary concerns among Whitaker Class II patients included scar-related issues (scar revision required in 3 patients, stretched scar in 1 patient) and skull contour irregularities (noted in 2 patients, one with associated temporal hollowing). All 5 patients classified as Whitaker Class IV had undergone secondary cranial surgery for aesthetic indications.

### Parent/patient-reported satisfaction

Parent-reported satisfaction with long-term head-shape outcome was high across the cohort. Seventy-five patients (82.4%) were completely satisfied and would not consider further head-shape surgery (Level I), 11 patients (12.1%) were mostly satisfied but might consider minor contouring if it were simple or low risk (Level II), and 5 patients (5.5%) were dissatisfied and actively seeking or desiring further surgery (Level IV). No patients were classified as Level III.

Among the 11 patients reporting Level II satisfaction, the main concerns included scar-related issues (stretched scar in 4 patients, scar revision desired in 3 patients), skull contour irregularities (bone ridging in 1 patient, skull irregularities in 1 patient, temporal hollowing in 1 patient), and residual head-shape concerns (1 patient). All 5 patients expressing dissatisfaction (Level IV) had undergone secondary aesthetic cranial surgery.

### Correlation between surgeon and parent/patient outcomes

**Fig. 5:**
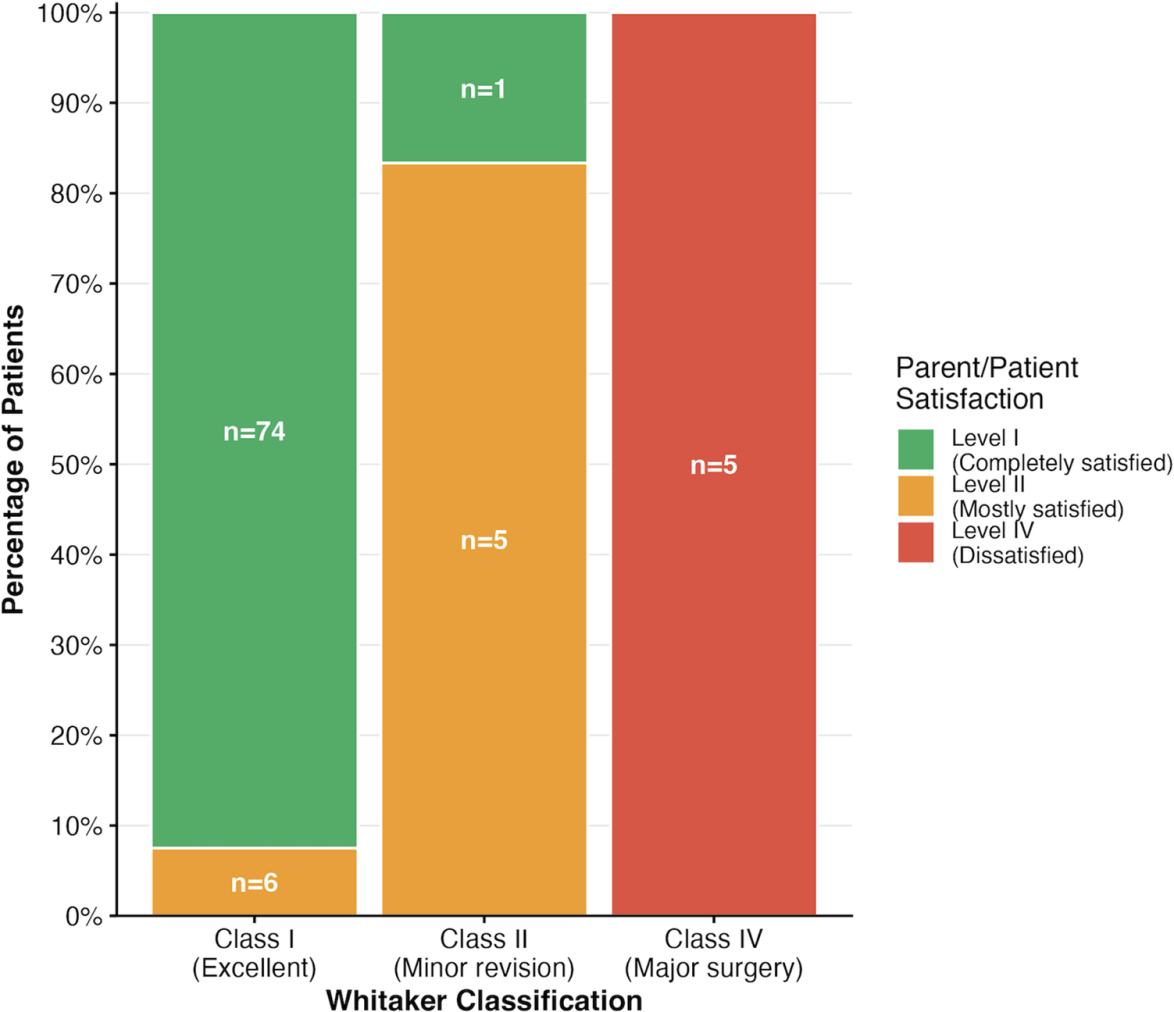
Correlation between surgeon-reported outcomes and parent/patient satisfaction. **a** Heatmap showing the distribution of patients across Whitaker Classification (surgeon assessment) and Parent/Patient Satisfaction levels. Numbers indicate patient counts in each category, with colour intensity representing the magnitude. **b** Stacked bar chart showing the percentage distribution of parent/patient satisfaction levels within each Whitaker classification category. Among patients with excellent surgical outcomes (Whitaker Class I, *n* = 80), 92.5% reported complete satisfaction. All patients with poorer surgical outcomes (Whitaker Class IV, *n* = 5) reported dissatisfaction, demonstrating strong concordance between professional and family assessments of long-term outcome

Analysis of the relationship between surgeon-reported Whitaker classification and parent/patient satisfaction revealed a strong concordance between professional assessment and family perception of outcomes (Fig. 5a). Among the 80 patients classified as Whitaker Class I, 74 (92.5%) reported complete satisfaction (Level I), while 6 (7.5%) were mostly satisfied (Level II), and none reported dissatisfaction. Of the 6 patients classified as Whitaker Class II, 1 (16.7%) was completely satisfied, 5 (83.3%) were mostly satisfied, and none were dissatisfied. All 5 patients classified as Whitaker Class IV reported dissatisfaction (Level IV, 100%).

The overall concordance between surgeon and parent/patient assessments was high (Fig. 5b), with excellent outcomes (Whitaker Class I) strongly associated with complete satisfaction (Level I), and poorer surgical outcomes (Whitaker Class IV) uniformly associated with dissatisfaction (Level IV). The intermediate category (Whitaker Class II) showed greater variability in parent satisfaction, though the majority remained satisfied overall. This pattern suggests that surgeon assessment of long-term aesthetic outcome using the Whitaker classification generally aligns well with parent and patient perception of head shape and satisfaction with surgical results.

## Discussion

In this study, we present long-term outcomes of spring-assisted cranioplasty for isolated, nonsyndromic sagittal synostosis in a large, single-institution cohort with follow-up extending into late childhood and adolescence. With a mean follow-up of approximately 10 years and a maximum follow-up of 15 years, this analysis provides one of the longest reported longitudinal assessments of this technique. The majority of patients remained free from secondary cranial surgery over the duration of follow-up, and both surgeon-reported and parent-reported outcomes demonstrated high levels of long-term satisfaction. Together, these findings suggest that spring-assisted cranioplasty offers durable cranial correction with sustained functional and aesthetic outcomes when applied within a structured multidisciplinary follow-up pathway.

A central challenge in evaluating surgical correction of sagittal synostosis lies in defining meaningful measures of long-term success. While early postoperative improvements in cephalic index are frequently reported following spring-assisted cranioplasty, it is increasingly recognised that cephalic index alone does not adequately capture the complexity of cranial shape or reliably predict long-term functional or aesthetic outcomes [25, 28]. More importantly, early morphologic change does not necessarily reflect durability of correction. From a clinical perspective, the need for secondary cranial surgery, particularly for raised intracranial pressure or persistent deformity, represents a more robust and patient-relevant endpoint [29]. By focusing on long-term reoperation rates and time-to-event analysis, the present study provides insight into the sustained effectiveness of spring-assisted cranioplasty beyond the early postoperative period.

One of the clinically relevant findings of this long-term analysis is the low incidence of raised intracranial pressure following spring-assisted cranioplasty. Only four patients (4.4%) developed clinically significant raised intracranial pressure requiring secondary surgical intervention during extended follow-up, meaning that more than 95% of patients with isolated, nonsyndromic sagittal synostosis remained free from ICP-related surgery up to 15 years after the index procedure. Importantly, all cases of raised intracranial pressure occurred several years after the initial correction, at 25, 38, 49, and 71 months postoperatively, corresponding to an age range of approximately 2 to 6.5 years. This timing corresponds to a period of continued brain growth and skull remodeling, making the development of raised intracranial pressure during early childhood biologically plausible. In this cohort, three of the four cases were identified through routine ophthalmological surveillance with detection of papilledema, highlighting the importance of structured follow-up and regular fundoscopy during this at-risk period. Beyond early childhood, the occurrence of new-onset raised intracranial pressure appeared to be uncommon. When considered in the context of published reports describing higher baseline rates of raised intracranial pressure in isolated sagittal synostosis [30, 31], these findings support the long-term safety of spring-assisted cranioplasty, while emphasizing the need for methodical, multidisciplinary surveillance during key phases of cranial growth.

Reoperation for aesthetic reasons was also uncommon in this cohort, with five patients (5.5%) requiring secondary cranial surgery for appearance-related concerns over extended follow-up. The majority of these procedures were undertaken to address persistent frontal bossing. Importantly, accumulating evidence over the past decade has demonstrated that frontal contour abnormalities, particularly frontal bossing, often improve with time as a consequence of ongoing craniofacial growth, including temporal muscle development and progressive frontal sinus pneumatization [32, 33]. This improved understanding has led to a more conservative contemporary approach, with greater emphasis on observation and delayed reassessment before considering fronto-orbital remodeling in patients treated with spring-assisted techniques. As the present cohort represents the initial 100 cases treated during the early adoption of this approach, most aesthetic reoperations occurred over 8 years ago, prior to widespread recognition of these growth-related changes. Consequently, the proportion of secondary aesthetic procedures observed in this historical cohort is likely higher than would be expected in current practice, where delayed decision-making has proven effective in reducing unnecessary reintervention.

An additional strength of this study lies in the parallel assessment of surgeon-reported and parent-reported long-term outcomes, allowing exploration of how professional evaluation aligns with family perception. Overall, a high degree of concordance was observed between surgeon-assigned Whitaker classification and parent-reported satisfaction, indicating that objective assessment of cranial shape generally reflects family experience over time. Notably, the majority of cases classified as Whitaker Class II were related to minor scar concerns rather than residual cranial deformity. Although the surgical scar is small and typically concealed within the hair-bearing scalp, it remained a source of concern for a subset of patients and families, accounting for fewer than 15% of the cohort. In most instances, observation and reassurance were recommended, and only three patients ultimately required minor scar revision. Importantly, these scar-related concerns were not associated with functional impairment or dissatisfaction with overall head shape, underscoring that intermediate outcome classifications often reflect preference-sensitive issues rather than substantive surgical shortcomings. Together, these findings highlight the importance of incorporating patient- and parent-reported perspectives into long-term follow-up and support a measured, shared decision-making approach when considering secondary interventions.

The long-term reoperation rate observed in the present cohort compares favourably with published series employing alternative surgical strategies for isolated sagittal craniosynostosis. Runyan et al. [34] reported equivalent reoperation rates and Whitaker outcomes between spring-assisted surgery and cranial vault remodeling at up to 12 years of follow-up, with spring-assisted surgery offering substantially lower operative time, blood loss, and hospital stay. Similarly, Windh et al. [19] in one of the earliest direct comparative studies, found comparable long-term cephalic index correction between spring-assisted cranioplasty and pi-plasty. More recently, a systematic review of endoscopic techniques reported pooled reoperation rates of approximately 3%, though with considerably shorter follow-up than the present series [35]. For open cranial vault remodeling, reported reoperation rates from large series range from approximately 9 to 11%, encompassing both syndromic and nonsyndromic cases [36]. The present cohort, comprising exclusively nonsyndromic isolated sagittal synostosis with follow-up extending to 15 years, demonstrates a 9.9% total secondary surgery rate, the majority of which reflects the early adoption period of this technique. Whilst direct comparisons across institutional series are inherently limited by differences in patient selection, follow-up duration, and the definition of reoperation, these data collectively suggest that spring-assisted cranioplasty achieves durable long-term correction broadly comparable to alternative approaches, while retaining the perioperative morbidity advantages of a minimally invasive strategy.

Surgical complications specific to spring-assisted cranioplasty warrant consideration. In the present cohort, no cases of spring migration, spring exposure, or wound dehiscence were recorded during the expansion phase. Dural breach is the most consequential intraoperative risk, mitigated in our technique by the routine use of a central craniectomy to decompress the sagittal sinus prior to parasagittal osteotomies and by meticulous dural dissection along planned osteotomy lines before bone cutting. Asymmetric expansion, which may result from incomplete extension of the osteotomies into the coronal and lambdoid sutures, was identified as an important technical pitfall during the early adoption phase and is now addressed by systematic intraoperative verification of osteotomy extent. Spring-related discomfort in the supine position, observed with opposing helix orientations in earlier cases, led to a standardised modification placing both helices anteriorly. Other reported complications across published series include surgical site infection and spring dislodgement, with rates of approximately 1–4% in contemporary series [14]. The two-stage nature of the procedure, requiring a planned return for spring removal at approximately 3 months, represents an additional consideration for families; however, spring removal is consistently performed as a day-case procedure with minimal morbidity.

This study has several important strengths, including a large, consecutive single-institution cohort, a standardized surgical technique, and a structured multidisciplinary follow-up pathway extending into late childhood and adolescence. The availability of long-term data allowed assessment of clinically meaningful endpoints, including secondary cranial surgery, surgeon-reported outcome, and parent-reported satisfaction. Nevertheless, the findings should be interpreted in light of certain limitations. The study is retrospective in nature, reflects the experience of a single high-volume centre, and lacks a contemporaneous control group treated with alternative techniques. In addition, the cohort represents the early adoption phase of spring-assisted cranioplasty, during which surgical decision-making and aesthetic thresholds have continued to evolve. Despite these limitations, the present data provide robust evidence that spring-assisted cranioplasty for isolated, nonsyndromic sagittal synostosis is associated with durable long-term outcomes, a low incidence of raised intracranial pressure, and high levels of patient and parent satisfaction when combined with methodical long-term surveillance. These findings support spring-assisted cranioplasty as a safe and effective component of contemporary sagittal synostosis management and underscore the importance of prolonged follow-up to guide individualized, growth-informed decision-making.

## Conclusion

Spring-assisted cranioplasty for isolated, nonsyndromic sagittal synostosis provides durable long-term outcomes, with a low incidence of secondary cranial surgery and sustained functional and aesthetic results extending into late childhood and adolescence. The majority of patients remained free from raised intracranial pressure, and both surgeon- and parent-reported outcomes demonstrated high levels of long-term satisfaction. These findings support spring-assisted cranioplasty for scaphocephaly as a safe and effective technique with robust long-term outcomes when undertaken within a structured, multidisciplinary craniofacial programme.

## Data Availability

The datasets generated and/or analyzed during the current study are available from the corresponding author upon reasonable request.
